# Glycerine in Phthisis

**Published:** 1858-01

**Authors:** J. H. Robinson

**Affiliations:** Jone, Ill.


GLYCERINE IN PHTHISIS.

JONE, ILL., Aug. 4th, 1857.

N. S. DAVIS, M.D.,—I have lately been necessitated to use *glycerine*, after the manner of your formula as given in the *Journal*, in tuberculosis. In every case where I have used it, the patient has complained of *pain* in the *stomach*, commencing with its use. Have you heard such complaint? If so, what occasions it? I am yours truly,

J. H. ROBINSON.

[We have prescribed glycerine almost daily for one or two years past, both in hospital and private practice, but have heard of no such complaint as above stated. We think it must be owing to the use of an impure article by our correspondent.—Ed. *Journal.*]

